# What Are the Factors That Influence Job Satisfaction of Nurses Working in the Intensive Care Unit? A Multicenter Qualitative Study

**DOI:** 10.1155/2023/6674773

**Published:** 2023-04-14

**Authors:** Gijs Hesselink, Floor Branje, Marieke Zegers

**Affiliations:** Radboud University Medical Center, Department Intensive Care Medicine, Nijmegen, Netherlands

## Abstract

**Aim:**

To explore and describe the factors that influence the job satisfaction of nurses working in the intensive care unit (ICU).

**Background:**

High turnover and dropout rates of nurses currently put pressure on the accessibility and quality of ICU care. Job satisfaction is an important predictor for turnover. However, there is little knowledge about the factors that enhance or frustrate the job satisfaction of ICU nurses.

**Methods:**

A qualitative descriptive study was conducted from March to July 2022. Semistructured interviews were held with 23 registered nurses who were purposively sampled from the ICU in four hospitals in the Netherlands. Interview transcripts were analyzed by using a thematic content analysis approach.

**Results:**

Six themes emerged: (1) being part of a solid team; (2) professional autonomy; (3) competence development; (4) appreciation of work by others; (5) work content; and (6) human resource management. Interviewees described the importance of being part of a team, having professional autonomy and opportunities to develop and remain challenged as a professional. In practice, these needs are often not met. Interviewees expressed their own role in meeting these needs by taking charge in situations, being eager to learn, and actively looking for ways to keep work attractive. Recognition and appreciation for their work are important catalysts for staying motivated. Monotonous work, poor leadership, and bureaucracy reduced their job satisfaction.

**Conclusion:**

Our findings provide deeper insight into a range of factors that influence the job satisfaction of ICU nurses and may also apply to nurses in other settings. Practical recommendations are given for keeping the nursing profession attractive for the current and future generation. *Implications for Nursing Management*. Findings emphasize the importance of optimizing nurses' work conditions by investing in their social embeddedness, professional autonomy, opportunities for competence development, and appreciation of work.

## 1. Background

Due to an increasing demand for healthcare and a growing shortage of nursing staff, healthcare services are increasingly experiencing difficulties in providing accessible and high-quality care on a continuous basis [1, 2]. Moreover, staff shortages can jeopardize patient safety, especially in clinical departments where highly qualified nurses are needed, such as in the intensive care unit (ICU) [3, 4].

Job satisfaction is an important predictor for the turnover (intention) of nurses [5–7], which in turn contributes to staff shortage. Job satisfaction can be defined as the degree to which individuals feel positive or negative about their job [8]. Others described job satisfaction as an attitude or emotional response to one's tasks as well as to the physical and social conditions of the workplace [9], or as a pleasurable emotional state resulting from one's job [10], which is often determined by the degree of motivation in doing job activities and the extent to which basic psychological needs at work are met, namely, autonomy, relatedness, and competence [11, 12]. High turnover rates among nurses in many countries show that job satisfaction among this group of professionals is under pressure [13–15]. A recent meta-analysis of 18 cross-sectional studies including ICU nurses from 23 countries showed that more than 27% had the intention to leave the ICU or the nursing profession [16]. In addition, the corona pandemic has taken its toll on the nursing workforce and increased the risk of drop out due to mental problems. Recent nationwide surveys among ICU nurses in the Netherlands showed that one in three are considering quitting their job [17, 18]. As a result of the growing shortage of ICU nurses, the workload increases, and this puts additional pressure on the remaining staff [18, 19].

Despite its predictive value on the turnover of nurses, insight into the factors that enhance or frustrate ICU nursing satisfaction lacks. A large body of literature focused on nursing satisfaction levels and contributing factors. However, reviews showed that studies including ICU nursing staff were performed more than a decade ago and generally used quantitative methods, which are insufficient for fully understanding the underlying factors for (dis)satisfaction [20, 21].

A better understanding of the factors that enhance or frustrate job satisfaction among ICU nurses, based on their daily experiences, is needed to find ways or support current initiatives for improving job satisfaction in order to retain nurses for the ICU profession. Moreover, this understanding could also be beneficial for managers outside the ICU setting dealing with high nursing staff shortage and turnover. Therefore, we conducted a qualitative study among ICU nurses to explore what factors influence job satisfaction in daily ICU practice.

## 2. Methods

### 2.1. Design, Setting, and Participants

This study used a qualitative descriptive design to understand the ICU nurses' perspectives on factors influencing job satisfaction. Registered nurses with a postgraduate qualification or specialized training in critical or intensive care nursing and employed (fulltime or part-time) by the ICU were eligible for participation. Unit or nurse team leaders were excluded as we wanted to gain insight into the perceptions of those who are primarily responsible for delivering bedside care and not from those who are (partly) responsible for creating and maintaining optimal working conditions for ICU nurses. The nurses were recruited at one academic and three regional hospitals in the eastern part of the Netherlands. Like in most other high-income countries, there is a shortage of specialized ICU nurses in the Netherlands which can impact nurses' job satisfaction. Moreover, the percentage intention to leave and the burnout prevalence among Dutch ICU nurses are fairly generalizable compared to other high-income countries [16, 22]. Therefore, we assumed that the insights on job satisfaction from this Dutch study sample would also be of value to the nursing audience outside the Netherlands. Purposive sampling was used to recruit a heterogeneous sample across a number of characteristics (age, work experience, and hospital/department) in order to obtain as many different perspectives as possible. Eligible candidates were identified through contact persons and interviewees themselves (snowball sampling) and then approached by e-mail to participate in the study.

### 2.2. Data Collection

After obtaining informed consent, 23 interviews were held between March and July 2022. The interviews were semistructured and conducted face-to-face at the participant's work location by one of the authors (FB). Interviews were held in a private room with a closed door to allow participants to talk openly about their job satisfaction. The interviewer, a registered hospital nurse following a two-year master program in Public Administration, was trained in conducting interviews prior to this study. Interviews were held using a guide with initial questions and probes (Supplement 1, interview guide). The interview guide was developed using sensitizing concepts found in the literature on determinants of job satisfaction and in discussion with researchers with a background in health and organizational sciences (GH, MZ). Sensitizing concepts, a starting point for much of qualitative research, give guidance when approaching a phenomenon or experience but do not prescribe what the researcher should see; rather, they suggest where a researcher might want to look and sensitize them to possible lines of inquiry [23]. Interviewees were encouraged in the interviews to substantiate their perceptions on the basis of concrete experiences. Interviews lasted between 21 and 54 minutes.

### 2.3. Data Analyses

All interviews were audio recorded, transcribed verbatim, and then systematically analyzed according to the principles of thematic content analysis [24]. The analysis started with reading and re-reading the first three transcripts to become familiar with and gain an overall sense of the content. Subsequently, FB generated initial codes by providing conceptual labels to relevant text passages representing an influencing factor. The generated codes were based on the data itself and driven by theoretical models on job satisfaction and work motivation [11, 12]. This resulted in the development of an initial codebook that, after being discussed and revised by GH and FB, acted as a blueprint for the coding of new transcripts. After the analysis of each new transcript, FB critically examined the list of codes for relevance, uniqueness, and formulation and made revisions when needed. Codes that referred to the same underlying concept were grouped into categories and then placed in overarching themes. In several rounds, codes, categories, and themes were discussed between two researchers (FB and GH) to reach agreement on structure, wording, and relevance. In case of disagreement, a third researcher (MZ) was consulted. Data collection and analyses stopped after no new findings emerged, and data saturation was reached. Illustrative quotes were selected to support the main findings. Data analyses were supported by using a qualitative data analyses software program (Atlas.ti Version 22). The themes, categories, and quotes were translated into English by GH and checked on spelling, grammar, and content by FB and MZ.

### 2.4. Ethical Considerations

The local medical ethical committee “CMO Arnhem-Nijmegen” approved the study (identification number: 2021–8156). Written informed consent was obtained prior to the interview. Participants were informed that they could refuse to answer any questions or withdraw from the study at any time. Participant confidentiality was ensured by deidentifying interview transcripts, and only research members had access to the data.

### 2.5. Rigor

The research was conducted and reported with accordance with the Standards for Reporting Qualitative Research (SRQR) to ensure study trustworthiness [25]. Transferability is supported by providing contextual information such as age, hospital type/work setting, and working experience in the ICU (Table 1). This allows the reader to determine if the findings apply to other similar populations. By frequently reflecting on the interviews and the meaning of data with a group of researchers with different professional backgrounds, we have tried to limit the influence of personal experiences and subjectivity in the research process and meet the requirements of reflexivity [26]. To ensure credibility, at the end of each interview, the conversation was summarized and interviewees were given the possibility to reflect and comment on the accuracy and validity of the obtained information.

## 3. Results

### 3.1. Participant Characteristics and Identified Themes

Demographic characteristics of the 23 participants are presented in Table 1. Most participants were female (65%) with a median age of 42 years (range 24–57). Most of the interviewees were experienced ICU nurses (median 15 years) and worked in the ICU of a university hospital (61%). Educational level was almost equally distributed. The analysis resulted in 21 categories from which six themes emerged. The themes, categories, and interview quotes are shown schematically in Supplement 2 (Supplement 2, Table with themes, categories, and interview quotes). The findings are discussed in more detail below for each theme.

### 3.2. Being Part of a Solid Team

All interviewees indicated the importance of *stable teams and team membership*. Being part of a close-knit team of colleagues makes it easier to give and receive professional and personal support, and to share joys and sorrows. It also provides more room for small talk and a pleasant collegial *atmosphere*. Interviewees argued that these aspects are important in their work where they are structurally dealing with emotional situations and work under pressure with a lot of responsibility. According to interviewees, the importance of working in a team became particularly clear during the corona pandemic. While some could fall back on the social ties and support within their team, this was missed by others due to constantly changing team compositions.

*We're dealing with complex and difficult work here. A lot of people don't make it anyway. And you need a very strong team for that, around you, to be able to handle all of that. [Nurse 1]*

### 3.3. Professional Autonomy

Experiencing autonomy—or the lack of it—also impacted the job satisfaction of many interviewees. With professional autonomy interviewees meant the ability and freedom to act independently and influence treatment decision based on their own professional nursing insights.

*The fact that I experience little autonomy affects my job satisfaction. (...) I also just want to have some autonomy and be able to determine things myself without a doctor always having to look over my shoulder, telling me things to do or having an opinion about my work. [Nurse 4]*

Interviewees experience a *varying degree of professional autonomy*. Many referred to the *work culture* in their ICU as an important factor limiting or increasing their autonomy. On the other hand, interviewees described that professional autonomy of nurses in their ICU is also strongly determined by nurses' *personal characteristics* such as assertiveness and working experience.

*We are too humble. We let others lead us too easily. This also applies to me. I don't always grab that autonomy when it's there. And why? I think that's because we lean back too much and then we start to complain that we don't have it while we don't take it either. [Nurse 14]*

### 3.4. Competence Development

Having *opportunities to develop and specialize* in the field of nursing and ICU care was also often cited as an important factor for job satisfaction. Interviewees referred to the importance of following education, visiting symposia, participating in working groups, and picking up areas of interest on specific care themes such as pressure ulcer prevention, and palliative and comfort care. Apart from enriching their day-to-day bedside work, interviewees indicated that these activities also help them to increase their career prospects. Interviewees indicated that competence development needs to be stimulated and facilitated by the hospital and ICU management providing sufficient budget and available time for abovementioned activities.

*The feeling that those opportunities are there and that they are offered from the department, that's reassuring to me. That you can develop further if you wanted to. [Nurse 10]*

Job satisfaction is also influenced by the presence or absence of a *learning mindset* of ICU nurses themselves, for example, by being open to new working methods and by approaching complex patient cases as a challenge and learning opportunity instead of a problem or burden.

*Finding joy in work is mainly about keep challenging yourself and being able to grown as a professional (…) For example, by having the right mindset when you are dealing with a complex patient case. [Nurse 16]*

Furthermore, *variation in job tasks and working with different patient categories* contributes to job satisfaction, according to most interviewees. On the contrary, performing monotonous work leads to boredom and dissatisfaction.

*I like doing things besides taking care for patients. For example, I participate in several committees. So variety of work is very important to me. If I had to do the same thing every day, day in, and day out. I would find that very boring. [Nurse 14]*

### 3.5. Appreciation of Work by Others

Interviewees derive great satisfaction from the *appreciation of their nursing care by patients and relatives*, for example through thank-you cards and the feedback they receive from colleagues in the post-ICU clinic. These forms of appreciations are important to them as it directly touches their core motive for being a nurse, namely to make a difference for someone in need of care. *Appreciation from supervisors and co-workers* via a compliment or a pat on the back were also mentioned as important drivers for job satisfaction.

*Of course everyone wants to be seen and appreciated. That's vital for enjoying work. Without that, you won't be seen and everything stops. [Nurse 6]*

According to interviewees, salary does not directly affect their job satisfaction. However, most of them do experience *an imbalance between the amount of their salary and the nature of their work* (e.g., irregular working hours, major responsibilities, and emotional impact). According to them, this imbalance contributes to a negative atmosphere of underappreciation and frustration in the workplace that, in turn, negatively affects their job satisfaction.

*When I compare my wage and the responsibilities in our work to other professions, I don't think it's very equally and fairly distributed. (…) It doesn't necessarily affect my job satisfaction though. I chose this job and I knew what I was getting into. I knew I wasn't going to sit in an office and paid tree times more than what I'm earning right now. (…) But because everyone is talking about that, especially after COVID, it does make you think about it and feel: ″Oh, what we are doing is not fully appreciated″. [Nurse 12]*

### 3.6. Work Content

Interviews show the positive and negative impact of ICU nurse activities on job satisfaction. In addition to providing patient care and *supervising nursing students*, many interviewees derive great satisfaction from family *counseling*. The impact of an ICU admission on family members—due to the uncertain course and unstable condition of most ICU patients and the limited communication possibilities with the patient—makes this guidance role very meaningful to them.

A couple of work activities were frequently mentioned as factors reducing job satisfaction. Performing *basic nursing tasks* such as cleaning and mobilizing ICU patients were regarded as necessary but less enjoyable, or at least, not the tasks for which they became ICU nurses and draw energy from. Furthermore, interviewees were frustrated by the *administrative tasks* they often regarded as unnecessary and time consuming. Moreover, the administrative work comes at the cost of activities they perceive more valuable and derive satisfaction from, namely, the care and attention for ICU patients, and their loved ones.

*Figuring out how to then get a certain bed or material to the department, for example. (...) Those are not the things that make my job more fun. It's mainly the extra effort and amount of paper work that makes it less attractive. [Nurse 21]*

Finally, some interviewees indicated that their job satisfaction has decreased since the more complex and specialized care is no longer provided in their hospital and work mainly consists of less complex and standard nursing care.

### 3.7. Human Resource Management

Several interviewees described how the active *involvement of managers* and their action-driven approach regarding problems in the ICU positively affected their joy in work. However, the majority experienced the opposite: upper management is often invisible, lacks empathy, and is unaware of what is going on in the workplace. Problems and concerns that exist at the work floor, for example, regarding workload and care-specific themes, are not sufficiently addressed. This experienced *gap between management and the work floor* frustrates interviewees. Especially, since in their view, the management is responsible for creating the conditions within which they can do their work well and with joy. Interviewees also described their frustration with *bureaucracy* in the hospital. The realization of promising quality improvement initiatives, developed bottom-up, are often hindered by the involvement, and formal approval needed from many parties.

*Practical ideas for improving care or tackling a problem in the ICU are often hindered by bureaucracy. Such practical initiatives have to pass a x-number of people and require formal approval. (...) In the beginning it motivated me, but at a certain moment I started to notice that nothing really happens. When new ideas are mentioned I tend to think: “Never mind, forget it.” [Nurse 16]*

Interviewees furthermore referred to the *availability of basic work facilities* at the workplace as factors influencing job satisfaction, such as sufficient parking space, healthy food and drink options, and a dedicated place in the ICU for professionals to destress and relax. Finally, the absence of self-rostering or having a little *influence in scheduling work shifts* was often mentioned as a factor reducing joy in work.

## 4. Discussion

This qualitative study provides a deeper understanding of the factors that positively and negatively influence job satisfaction of ICU nurses. In accordance with the previous literature [20, 21], the findings show that job satisfaction is largely determined by the extent to which certain needs are met. ICU nurses indicated specific need for (1) working with the same colleagues on a permanent basis to ensure interpersonal bonding and emotional support, (2) freedom to act independently and the ability to influence treatment decisions based on their nursing expertise, and (3) opportunities and incentives to remain challenged and grow as a professional. The interviews showed that—due to external factors such as a working culture focused on accountability, inadequate management of human resources, and the corona pandemic—these needs are often not met in daily practice, thereby reducing the job satisfaction of ICU nurses. In addition to the external factors, ICU nurses expressed their own role in meeting these needs, for example by taking charge in situations, being eager to learn, and looking actively for ways to keep work attractive. Although hospital nurses are being regarded as the main person responsible in the healthcare team when it come to an holistic understanding of what the patient needs, this does not always coincide with the freedom to act accordingly [27].

Our findings also show that job satisfaction among ICU nurses stems from their intrinsic motivation to be of value to ICU patients and their loved ones. Recognition and appreciation for their work—by patients, relatives, colleagues, and managers—are in that respect important motivational drivers for ICU nurses. Other studies already addressed the importance of these factors for other nursing groups [28, 29]. Furthermore, the interviews showed that job satisfaction in the ICU is reduced by monotonous work, inefficient administrative work, poor leadership, and bureaucracy. Contrary to several other studies [30, 31], external drivers such as salary and workload seem to have less direct influence on job satisfaction. Existential motives prevail. Drennan et al. [32] reported how one nurse felt: ″Pay is important, but most people don't come into nursing expecting high salaries—they have other motivations.″ However, the perceived imbalance between salary level and the nature of their work does contribute to a widespread feeling of undervaluation among ICU nurses and thus indirectly influences their job satisfaction.

### 4.1. Recommendations

This study shows that increasing job satisfaction requires a joint effort. On the one hand from policy-makers, managers, and ICU physicians by creating an attractive work environment, and on the other hand from ICU nurses themselves by finding ways to keep their profession attractive. Nurses who are proactive and show leadership experience greater job satisfaction and are less likely to leave the profession [33]. Based on our findings and inspired by previous papers [34, 35], we listed several recommendations for practice across seven domains which may help to increase job satisfaction among ICU nurses and thereby retain them for the ICU (Table 2). Although the list focuses on ICU nurses, many of the recommendations we propose here may equally apply to the retention of nurses in other healthcare settings as nurse job dissatisfaction and high turnover are common in other healthcare settings as well [36, 37].

### 4.2. Limitations

Our study has several limitations. First, although we purposively sampled participants to ensure diversity of opinions and experiences, our study population was relatively small and consisted of only Dutch ICU nurses. Most participants worked in an academic setting and had several years of work experience. Findings may therefore not be representative for the entire ICU nursing workforce including those who are less experienced and working in other healthcare systems and settings (e.g., with other nurse-bed ratios, responsibilities, wages, or incentives for personnel). Our findings may also be influenced by country-specific nurses' perceptions of role responsibilities and views on professional autonomy [38, 39], which may limit the transferability of the findings to countries with a different cultural context.

Nevertheless, many of the factors found are common phenomena in the hospital setting [20, 21], that most likely also occur in other ICU settings regardless of the type of health care system or hospital. Therefore, we believe that our findings are likely to be recognizable to ICU nurses outside the Netherlands. The results are thus a starting point for further understanding, validation (using both quantitative and qualitative methods on an international scale), and improvement on this theme. Second, job satisfaction is a multidimensional concept that lacks a clear and uniform definition [40]. Despite the use of a working definition in the interviews and in the data analysis, participants may have confused job satisfaction with related concepts such as mental and psychosocial well-being which may have affected our data. Finally, in qualitative research, the researchers' background plays an important role in data collection and analyses, which can be both a strength and a limitation. The nursing background of the main researcher (FB) may have influenced the collection and interpretation of the data.

## 5. Conclusion

This study contributes to the increased understanding of factors that currently influence job satisfaction among ICU nurses and shows the importance of (support for the development of) nurse leadership as a mean to improve job satisfaction among ICU nurses. Although future research is warranted to extrapolate the findings on a larger scale, we believe our findings are valuable for ICU health professionals, policy-makers, and managers in a variety of hospitals and countries in their efforts for keeping this profession attractive for the current and future generation of ICU nurses and keeping ICU care sustainable.

## Figures and Tables

**Table 1:** Participant characteristics (*n* = 23).

Characteristics	Score
Gender	
Female, *n* (%)	15 (65.2)
Male, *n* (%)	8 (34.8)
Working experience as an ICU nurse, median (range)	15 (2–32)
Age in years, median (range)	42 (24–57)
Age category	
24–30 years, *n* (%)	4 (17.4)
31–40 years, *n* (%)	6 (26.1)
41–50 years, *n* (%)	7 (30.4)
>50 years, *n* (%)	6 (26.1)
Hospital type	
Academic, *n* (%)	14 (60.9)
Regional, *n* (%)	9 (39.1)
ICU = intensive care unit	

**Table 2:** Recommendations for policy-makers, managers, and health professionals to improve job satisfaction of ICU nurses.

Recognition and appreciation	(i) Pay attention to what is performed well by ICU nurses, and tell them where their specific knowledge and skills have made the difference
	(ii) Value and facilitate the (provision of) feedback of former ICU patients and relatives (e.g., through aftercare clinic or mirror talks) so that ICU nurses are better informed of the value of their work

Leadership	(i) Provide leadership and mentorship training for team leaders and senior nurses so that they are able to perform their roles well (e.g., visible, accessible, empathetic, and action-oriented)
Social bonding and team spirit	(i) Provide solid team structures; limit constant personal changes within the teams
	(ii) Provide adequate means to promote social contact among colleagues (e.g., budget and space for social activities)
Work-life balance	(i) Promote self-care and provide ways for recuperation from work (e.g., sufficient breaks, healthy food and drinks, space for relaxation and leisure time)
	(ii) Offer nursing shift flexibility and choice by the use of a self-rostering system

Autonomy and role	(i) Encourage ICU nurses to act independently (within set boundaries) and express confidence in doing so
	(ii) Encourage the active participation of ICU nurses in treatment decisions. This requires a work environment in which all professional opinions are valued and taken seriously

Professional development	(i) Coach ICU nurses in their individual career aspirations and prospects within the ICU
	(ii) Provide budget and time for ICU nurses to follow courses and training programs and visit symposia/congresses
	(iii) Encourage ICU nurses to work on quality improvement on a certain topic of interest (e.g., participation in a committee)
	(iv) Create possibilities for job variation (e.g., via nurse exchange programs between ICUs in the region) and job-sharing (e.g., working partly in the ICU and in the emergency department or as an EMT/ambulance nurse)

Administrative tasks	(i) Review administrative duties of ICU nurses and eliminate those that do not contribute to quality improvement or are not necessary for accountability purposes

ICU = intensive care unit; EMT = emergency medical technician.

## Data Availability

The data (de-identified) that support the findings of this study are available from the corresponding author upon reasonable request.

## Supplementary Materials

Supplementary Materials Supplementary 1: interview guide. Supplementary 2: table with themes, categories, and interview quotes.
