# Supplementary material for: What Are the Factors That Influence Job Satisfaction of Nurses Working in the Intensive Care Unit? A Multicenter Qualitative Study
Source: J Nurs Manag. 2023 Apr 14;2023:6674773. doi: 10.1155/2023/6674773 (PMC11919104; doi:10.1155/2023/6674773)
Supplement: Supplementary Materials — Supplementary 1: interview guide. Supplementary 2: table with themes, categories, and interview quotes. [file 6674773.f1.zip › Supplement 1 Interview guide.docx]

**Supplement 1. Interview guide**

**Introduction**

Short explanation of study aim, background and processing of data.

Request permission for audio recording.

**Demographics**

Age?

Education?

Years of working experience as ICU nurse?

**Initial questions**

1. What does job satisfaction mean to you?

2. If you would rate your level of job satisfaction on a scale from 1 (lowest) to 10 (highest), what number would this be, and why?

3. What do you like the most in your work as an ICU nurse (gives you energy, motivates you, et cetera)?

- *How come?*
- *Examples?*
- *How does this affect you as a person/professional?*

4. What do you dislike the most in your work as an ICU nurse (gives you energy, motivates you, et cetera)?

- *How come?*
- *Examples?*
- *How does this affect you as a person/professional?*
- *How do you cope with this?*

5. What else has a positive/negative influence on your job satisfaction? Can you tell me something about this/ relate this to certain experiences?

*[Think of: work-private balance, workload, autonomy, competence development, collaboration with colleagues/team atmosphere, personal characteristics, salary, management style, administrative work]*

**Summary and ending**
