# Supplementary material for: What Are the Factors That Influence Job Satisfaction of Nurses Working in the Intensive Care Unit? A Multicenter Qualitative Study
Source: J Nurs Manag. 2023 Apr 14;2023:6674773. doi: 10.1155/2023/6674773 (PMC11919104; doi:10.1155/2023/6674773)
Supplement: Supplementary Materials — Supplementary 1: interview guide. Supplementary 2: table with themes, categories, and interview quotes. [file 6674773.f1.zip › Supplement 2 Table with themes, categories and interview quotes.docx]

**Supplement 2. Table with themes, categories and interview quotes**

| **Theme** | **Category** | **Interview quotes** |
| --- | --- | --- |
| Being part of a solid team | Stable teams and team membership | *Feeling comfortable in that team. That you just feel like: I fit in here, the people who work here understand me and I understand them. (...) I feel supported by them when things get tough. They know what's going on privately and I feel safe and free enough to tell them things. [Nurse 4]* |
|  |  | *We're dealing with complex and difficult work here. A lot of people don't make it anyway. And you need a very strong team for that, around you, to be able to handle all of that. [Nurse 1]* |
|  |  | *Colleagues are a safe base for sure. (...) that you know what to expect from each other. And – when you start with a shift together – having already that feeling of: ″Oh, this will be okay no matter what happens″. [Nurse 5]* |
|  | Good social atmosphere | *Being surrounded by a nice group of colleages is very important to me. That you’re able to make jokes and having a good laugh. That’s the case in my team. We have a lot of fun together. That’s the icing on the cake in my work so to speak. [Nurse 20]* |
| Professional autonomy | Varying degree of autonomy | *The fact that I experience little autonomy affects my job satisfaction. (...) I also just want to have some autonomy and be able to determine things myself without a doctor always having to look over my shoulder, telling me things to do or having an opinion about my work. [Nurse 4]* |
|  |  | *Right now we are not allowed to decide a lot of things ourselves (...) and also when it comes to our voice in decision-making, both regarding the organization of ICU care and the treatment of patients. These things reduce my job satisfaction. [Nurse 2]* |
|  | Work culture | *You have to be empowered here and dare to speak up. They [other nurses and physicians] accept and even respect that. I can imagine that some people, due to their personality, find this difficult. Autonomy is not given to you. (…) That a doctor says, ″What do you think?″ That's rare. [Nurse 22]* |
|  |  | *We don't have to constantly seek approval for everything. That's just how everyone was raised here (...) it's just culture. [Nurse 1]* |
|  | Personal characteristics | *We are too humble. We let others lead us too easily. This also applies to me. I don’t always grab that autonomy when it’s there. And why? I think that’s because we lean back too much and then we start to complain that we don't have it while we don't take it either. [Nurse 14]* |
|  |  | *You have to dare to take it, this autonomy. And that does differ for someone who just graduated. (...) You can play with that a little more when you're experienced. That's really fun, because you start doing what you think is important. [Nurse 21]* |
| Competence development | Opportunities to develop and specialize | *What still frustrates me are the limited career expanding opportunities. (...) So, you become an ICU or ED nurse or you go to the EMS. And then it kind of stops. [Nurse 14]* |
|  |  | *The feeling that those opportunities are there and that they are offered from the department, that's reassuring to me. That you can develop further if you wanted to. [Nurse 10]* |
|  |  | *Attending professional courses makes the job much more fun. I'd like a little more of this. A number of us have become self-employed. They are able to follow e-learning, for example on cardiology. [Nurse 19]* |
|  | Learning mindset | *Finding joy in work is mainly about keep challenging yourself and being able to grown as a professional (…) For example, by having the right mindset when you are dealing with an complex patient case. [Nurse 16]* |
|  | Variation in job tasks and patient categories | *I like doing things besides taking care for patients. For example, I participate in several commitees. So variety of work is very important to me. If I had to do the same thing every day, day in and day out. I would find that very boring. [Nurse 14]* |
|  |  | *I don't think you are challenged enough if you only provide ICU care. I need a certain 'trigger' to...if something comes very naturally to me, then it becomes sort of automatic. And if I think something is important, it's that care should never become an automatism. [Nurse 7]* |
|  |  | *It’s a nice patient category to care for, but it's mostly the same. At some point, one start to think about that when you work there [in the ICU department] for a number of years. [Nurse 15]* |
| Appreciation of work by others | From patients and relatives | *I like the signs of grattitude and tokens of appreciation from patients or family members. I'm also very happy with the staff of the ICU aftercare clinic who forward kind messages now and then. (...) I really like that. [Nurse 5]* |
|  |  | *Hearing something back from patients or family members. Yeah, that’s great. I really love that. [Nurse 6]* |
|  |  | *When the patient is finally on the ward, he has no idea who took care of him in the ICU. So you do get credit from family members. I often have a good relationship with them and that's nice. You notice that they are glad you are there. (...) That's whom you are doing it for, so to speak.* |
|  | From supervisors and co-workers | *Of course everyone wants to be seen and appreciated. That’s vital for enjoying work. Without that, you won't be seen and everything stops. [Nurse 6]* |
|  |  | *Some recognition for that you're doing a good job. I think everyone is sensitive to compliments and I'm sensitive to that as well. [Nurse 4]* |
|  | Perceived disbalance between salary and the nature of ICU work | *When I compare my wage and the responsibilities in our work to other professions, I don't think it's very equally and fairly distributed. (…) It doesn't necessarily affect my job satisfaction though. I chose this job and I knew what I was getting into. I knew I wasn't going to sit in an office and paid tree times more than what I’m earning right now. (…) But because everyone is talking about that, especially after COVID, it does make you think about it and feel: ″Oh, what we are doing is not fully appreciated″. [Nurse 12]* |
|  |  | *Healthcare is becoming more complex and more is expected of us. (...) The moment I have bad night and administer a wrong medication dosage, this may have irreversible lethal consequences. The moment a manager makes a wrong choice this could have at worst negative financial consequences. Consequences that sometime in the future can be fixed. (…) I do feel a little undervalued, for sure. (...) and we have to work really hard (...) long and irregular hours...you are always expected to be there. [D9]* |
| Work content | Counseling | *In fact, throughout my whole career I've really been getting a lot of satisfaction from interacting with and taking good care of patients and their loved ones. [Nurse 3]* |
|  |  | *You are doing work that really matters...for patients at the most vulnerable time in their lifes (...) but also for family members. [Nurse 15]* |
|  |  | *Taking care of family members is also very important (...) this aspect of work motivates me and gives me energy. (...) Most patients in the ICU are connected to a ventilator whichs makes communication very difficult or even impossible if they are also kept asleep. Especially in these cirucmstances I find it important to be in contact with the family and provide personal attention and support. [Nurse 15]* |
|  | Student supervision | *Just give me a student whom I can learn the intricacies of the profession. Yes, that makes my day. [Nurse 1]* |
|  |  | *In the ICU we have a lot of young graduates with almost no nursing experience, because the vacancies are just hard to fill. (…) It's always better, I think, to just learn the nursing profession on the regular wards, because that's also where you really learn to develop that clinical view and reasoning. (...) It demands more of us, because you actually have to teach them the basic skills and procedures. [Nurse 12]* |
|  | Basic nursing tasks | *I don't like washing people. I don't find it interesting to drag people into their chairs, so to speak. I do it all, because it’s part of the job, but it doesn't make me happy. [Nurse 1]* |
|  |  | *Washing and cleaning the same patient six times in one day doesn't make me happy, but it's just part of the job. I don't think about it too much either. (...) Just give me the METs, post-ICU consults for complex patients at the ward and the acute ICU-admissions. That's always more fun than cleaning up diarrhea. [Nurse 5]* |
|  | Administrative tasks | *Figuring out how to then get a certain bed or material to the department, for example. (...) Those are not the things that make my job more fun. It's mainly the extra effort and amount of paper work that makes it less attractive. [Nurse 21]* |
|  |  |  |
|  | Less complex and standard nursing care | *Major surgery has actually left the hospital. As a result, the ICU-patient population has changed (...) and the ED became our main supplier of patients. (...) This is an important loss. So if you look at the job satisfaction...for me it decreased because of this. [Nurse 7]* |
|  |  | *Specific patient categories are disappearing in our ICU. We don't see complex neuropatients anymore. (...) Very occasionally, but then it often goes quickly to Hospital X. That's a pity, because that's also an important aspect that reduces job satisfaction. [Nurse 16]* |
|  |  | *I don't get a lot of satisfaction for taking care of three [patients with] intoxications per week. Patients that are gone within a day. That really has to do with the patient category what is here in the ICU. [Nurse 12]* |
| Human resource management | Involvement of managers | *She really involves us in decisions about organization aspects and way to improve the quality of care. You can say anything to her. She also doesn't hesitate to say when things are not going well or if there is something important she notices. (...) Yes, she is very important for my job satisfaction. I also think that's why none of us left the ICU after the corona pandemic. [Nurse 20]* |
|  |  | *Our team leaders could sometimes be a little more empathetic, especially since they know what it’s like working as an ICU nurse in daily practice. (...) I often ask myself: ″Why is he/she reacting so cool and indecisive?″ Well, that is disturbing for your job satisfaction. [Nurse 21]* |
|  | Gap between management and work floor | *They make decisions that affect my job satisfaction. These decisions come largely from the hospital board of executives, not from our department. (…) You just notice that they are very far from the work floor. They really have no idea and sometimes make decisions that make me think: ″Guys, join us in the ICU for one day″. [Nurse 22]* |
|  |  | *I don't see those people [executives], I don't speak to them. (…) They do not talk to the nurses, but in the meanwhile they do determine everything. [Nurse 1]* |
|  | Bureaucracy | *Practical ideas for improving care or tackling a problem in the ICU are often hindered by bureacucracy. Such practical initiatives have to pass a x-number of people and require formal approval. (...) In the beginning it motivated me, but at a certain moment I started to notice that nothing really happens. When new ideas are mentioned I tend to think: ″Never mind, forget it″. [Nurse 16]* |
|  |  | *I notice that we are not moving forward here in the ICU. Improvement initiatives remain stuck in the development phase. (...) the moment you have a subject that you want to improve, for example resuscitation training or other small things, they almost don't get off the ground, because they have to be discussed and require approval at several levels. [Nurse 15]* |
|  | Availability of basic work facilities | *The fresh fruit on the table in the staff room, I love it! But at the same time we lose our lockers, have nowhere to park our car and sometimes can't even park our bike. This could be better.. [Nurse 9]* |
|  | Influence in scheduling work shifts | *We don’t work with self-rostering. So our work schedules are often suboptimal and irregular. This really frustrates me. [Nurse 20]* |
|  |  | *I really dislike the early shifts that are scheduled for me. That's why I find self-rostering so important (...) to be able to have more control over my work relative to my social life, which is also very important to me. [Nurse 23]* |
| ICU = Intensive Care Unit; ED = Emergency Department; EMS = Emergency Medical Service; MET= Medical Emergency Team. | | |
